# MetaboLights: towards a new COSMOS of metabolomics data management

**DOI:** 10.1007/s11306-012-0462-0

**Published:** 2012-09-25

**Authors:** Christoph Steinbeck, Pablo Conesa, Kenneth Haug, Tejasvi Mahendraker, Mark Williams, Eamonn Maguire, Philippe Rocca-Serra, Susanna-Assunta Sansone, Reza M. Salek, Julian L. Griffin

**Affiliations:** 1 European Bioinformatics Institute (EMBL-EBI), Wellcome Trust Genome Campus, Hinxton, Cambridgeshire CB10 1SD UK; 2 Oxford e-Research Centre, University of Oxford, Oxford, UK; 3 Elsie Widdowson Laboratory, Fulbourn Road, Cambridge, CB1 9NL UK; 4 Department of Biochemistry, University of Cambridge, Cambridge, CB2 1QW UK

**Keywords:** Metabolomics, Databases, ISA-Tab, ISA commons

## Abstract

Exciting funding initiatives are emerging in Europe and the US for metabolomics data production, storage, dissemination and analysis. This is based on a rich ecosystem of resources around the world, which has been build during the past ten years, including but not limited to resources such as MassBank in Japan and the Human Metabolome Database in Canada. Now, the European Bioinformatics Institute has launched MetaboLights, a database for metabolomics experiments and the associated metadata (http://www.ebi.ac.uk/metabolights). It is the first comprehensive, cross-species, cross-platform metabolomics database maintained by one of the major open access data providers in molecular biology. In October, the European COSMOS consortium will start its work on Metabolomics data standardization, publication and dissemination workflows. The NIH in the US is establishing 6–8 metabolomics services cores as well as a national metabolomics repository. This communication reports about MetaboLights as a new resource for Metabolomics research, summarises the related developments and outlines how they may consolidate the knowledge management in this third large omics field next to proteomics and genomics.

## Introduction

Metabolomics has become an important phenotyping technique for molecular biology and medicine. It assesses the molecular state of an organism or collections of organisms through the comprehensive quantitative and qualitative analysis of all small molecules in cells, tissues, and body fluids. Metabolic processes are at the core of physiology. Consequently, metabolomics is ideally suited as a medical tool to characterize disease states in organisms, as a tool for assessment of organisms for their suitability in, for example, renewable energy production, or for biotechnological applications in general. In addition application of metabolomics in environmental science, toxicology, food and medical industry is well established, growing and documented. Metabolomics studies generate large amounts of analytical data (Giga- to Terabytes depending on the size of the study) and therefore impose significant challenges for biomedical and life science e-infrastructures to cope with such data volumes and ensure that the data are captured, stored and disseminated based on open and widely accepted community standards. Years after the first standardisation exercises (Fiehn et al. 2007; Taylor et al. 2008), metabolomics is now reaching the state of a mature analytical technique as indicated by the establishment of 6–8 Regional Comprehensive Metabolomics Resource Cores (RCMRCs) by the NIH in the United States (http://grants.nih.gov/grants/guide/rfa-files/RFA-RM-11-016.html). In addition, we are now facing a rich ecosystem of specialised metabolomics databases, such as (Wishart et al. 2007; Kopka et al. 2005; Smith et al. 2005; Skogerson et al. 2011) as well as the first general metabolomics repositories (http://www.ebi.ac.uk/metabolights) and databases emerging. In Europe, the COSMOS consortium of 14 leading laboratories in metabolomics will begin its work on standards, data management and dissemination in metabolomics. Here, we outline these developments and show how they may consolidate the knowledge management in this third large omics field next to proteomics and genomics.

## MetaboLights: a cross-species repository for metabolomics experiments

The European Bioinformatics Institute (EMBL-EBI) has recently launched MetaboLights, a database for metabolomics experiments and the associated metadata. It aims to become the first comprehensive, cross-species, cross-platform metabolomics database maintained by one of the major open access data providers in molecular biology. The EBI ensures long-term stability and maintenance of the resource. Deposited datasets are assigned a stable identifier of the form MTBLS1 (the first dataset ever deposited in MetaboLights). These identifiers, like other stable identifiers in bioinformatics, can be used to mark datasets in publications or merge data in systems biology applications.

**Fig. 1:**
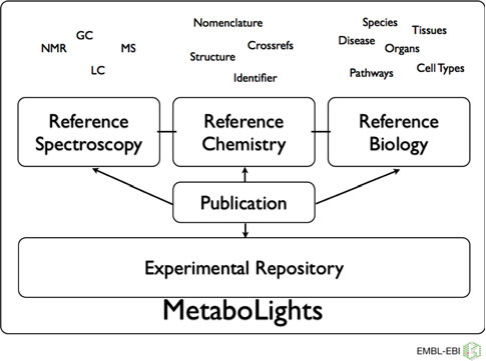
MetaboLights general outline with repository and reference layer. The reference layer is work currently in progress

**Fig. 2:**
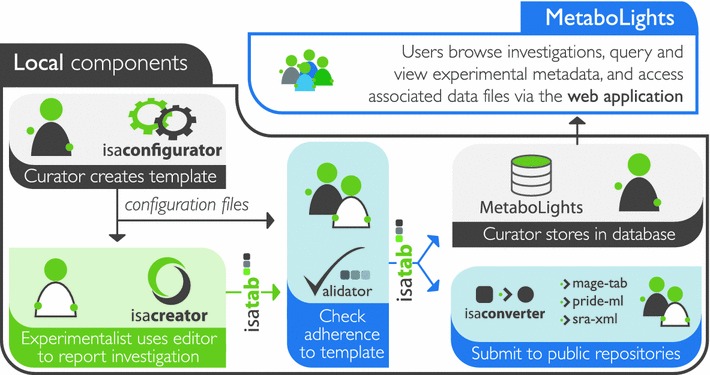
MetaboLights data submission workflow

Like all other EBI resources, the MetaboLights database is completely open to the public, including open access to the data. Data are made available in publicly accepted open standards compliant with community standards (BioSharing: http://biosharing.org/standards_view), including Minimum Information for Biological and Biomedical Investigations (MIBBI) checklists (Taylor et al. 2008). The software is open source and adheres to the promotion of open source file formats, such as mzML and nmrML. MetaboLights will ultimately consist of a reference later on top of the repository layer. The reference layer will contain information about individual metabolites and their chemical, analytical and biological properties. The repository later, which has been launched and is fully operational, contains primary research data from published metabolomics studies, annotated with meta data (Fig. 1). One of the main submission channels for MetaboLights is the ISA Tools Suite (Fig. 2) (Sansone et al. 2012).

**Fig. 3:**
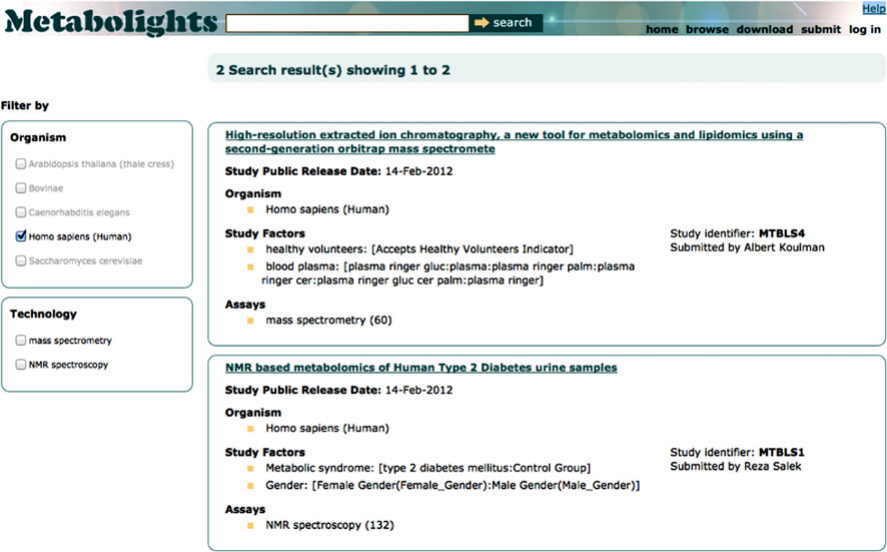
MetaboLights search results

MetaboLights is not intended to replace specialist resources for Metabolomics. Rather, it will build on prior art and collaborate. We are dedicated to close collaboration with all major parties involved in the creation of this prior art, such as the Metabolomics Society, Metabolic Profiling Forum (Metabomeeting) and the Metabolomics Standards Initiative (MSI). MetaboLights aims at formal data sharing agreements with major resources such as the Human Metabolome Database, the Golm Metabolome Database and the Rikken Metabolomics Platform. Currently we house a selection of experimental raw data and their associated metadata for different platforms such as NMR, GC-MS and LC-MS (Fig. 3). The repository layer is generally open to any data that was used in a metabolomics study. That could include, for instance, flux data (temporal measurements with ^13^C), spatial maps, and IR and Raman fingerprint data.

### Call for submitting data

MetaboLights is now ready for receiving metabolomics datasets. We have, for example, recently received the validation dataset measured by O’Callaghan *et al*. for validating their PyMS software (O’Callaghan et al. 2012). We think that this is the way forward for sharing gold standard datasets for validating metabolomics software. Generally, we hope, and will work towards this with journal editors, that the submission of datasets used to justify findings in publications will be submitted to the MetaboLights or one of the emerging collaborating repositories. Interested readers are encouraged to go to http://www.ebi.ac.uk/metabolights/presubmit and submit their data. The MetaboLights team is happy to assist in this process.

## Conclusion and outlook

Here, we have reported the publication of MetaboLights, the first cross-species, cross-platform metabolomics database maintained by one of the major open access data providers in molecular biology. MetaboLights lives at http://www.ebi.ac.uk/metabolights. For their convenience, readers can use the URL’s metabolights.org, metabolights.net and metabolights.eu. In October, the European COSMOS (COordination of Standards in MetabOlomicS) consortium will start its work on metabolomics data standardization, publication and dissemination workflows. It is the aim of COSMOS to develop efficient policies to ensure that metabolomics data are

1. Encoded in open standards to allow barrier-free and widespread analysis.
2. Tagged with a community-agreed, complete set of metadata (minimum information standard).
3. Supported by a communally developed set of open source data management and capturing tools.
4. Disseminated in open-access databases adhering to the above standards.
5. Supported by vendors and publishers, who require deposition upon publication
6. Properly interfaced with data in other biomedical and life science e-infrastructures (such as ELIXIR, BioMedBridges, EU-OPENSCREEN and BBMRI).

COSMOS will also strive to harmonize the European agenda with efforts in US, where the NIH is establishing 6–8 metabolomics services cores as well as a national metabolomics repository. Together with similar initiatives in Australia, Japan and hopefully more emerging over time, this opens the door for a global network of metabolomics data collection, exchange and dissemination.
